# Unplanned Re-Hospitalization Among Older Persons Results in Loss of their Independence

**DOI:** 10.1093/geroni/igaa057.3355

**Published:** 2020-12-16

**Authors:** Brandon Qualls, Hiwot Seyoum, Tammy Walker, Mary Carey

**Affiliations:** 1 University of Rochester Medical Center, Rochester, New York, United States; 2 Highland Hospital, Rochester, New York, United States

## Abstract

The Centers for Medicare & Medicaid Services defines a hospital readmission as an inpatient stay that begins within 30 days of the discharge date of an index admission, to the same or a different hospital. The aims of the study were to analyze the recurrent readmissions of older persons admitted to a community hospital with diagnoses of: Chronic Obstructive Pulmonary Disease (COPD), Pneumonia (PNA), and Congestive Heart Failure (CHF). Based on the results, we will develop additional strategies that can be used to reduce the rate for hospital readmission for older patients. A retrospective chart review of hospitalizations was conducted. Among 30 readmissions, the mean age was 79.5±14. The index disposition was distributed among three destinations: self-care (27%), home health organization (40%), and to skilled nursing home (33%). Most of the readmissions were for CHF (27%), COPD (10%), and PNA (13%), the only other large category include respiratory failure (10%). The readmission disposition was different from the index disposition: self-care (7%), home health organization (47%), and to skilled nursing home (20%). After hospital readmission within 30 days, older persons were more likely to be discharged to home health care organization than self-care, p<0.05. Actions that can be taken by hospitals to reduce 30-day readmissions to maintain older person independence include: clinical readiness of patients for discharge, proper infection prevention techniques, reconciling medications, good communication, and adequate patient education. This study reports that that older persons are at higher risk for unplanned hospital re-admissions and often lose their independence.

